# The unintended outcome: a retrospective cross‐sectional study using a urine lateral flow assay to detect ART use reveals non‐disclosure of taking ART in South Africa's public health system

**DOI:** 10.1002/jia2.26515

**Published:** 2025-06-23

**Authors:** Nsika Sithole, Indira Govender, Matthew Spinelli, Theresa Smit, Siyabonga Cibane, Mlungisi Zwane, Njabulo Phakathi, Meighan Krows, Busisiwe Nkosi, Janet Seeley, Ruanne V. Barnabas, Mark J. Siedner, Mosa Moshabela, Connie Celum, Alison Grant, Monica Gandhi, Adrienne E. Shapiro

**Affiliations:** ^1^ Africa Health Research Institute Somkhele South Africa; ^2^ London School of Hygiene & Tropical Medicine London UK; ^3^ Division of HIV, Infectious Diseases, and Global Medicine University of California San Francisco San Francisco California USA; ^4^ Department of Global Health University of Washington Seattle Washington USA; ^5^ University of Toledo Toledo Ohio USA; ^6^ University of KwaZulu‐Natal Durban South Africa; ^7^ Division of Infectious Diseases Massachusetts General Hospital Boston Massachusetts USA; ^8^ Harvard Medical School Boston Massachusetts USA; ^9^ Division of Allergy & Infectious Diseases Department of Medicine University of Washington Seattle Washington USA

**Keywords:** differentiated service delivery, HIV care continuum, HIV, TB, undisclosed ART, urine lateral flow assay

## Abstract

**Introduction:**

Differentiated service delivery (DSD) models for HIV and tuberculosis (TB) care prioritize efficient resource allocation and targeted interventions, and benefit from accurate assessment of patients’ antiretroviral therapy (ART) pill‐taking status. Accurate ART use identification is essential for ensuring proper care transition services rather than unnecessary initiation. A point‐of‐care urine tenofovir (TFV) assay may identify undisclosed ART use in settings with high rates of TB and HIV coinfection.

**Methods:**

A cohort of people living with HIV (PWH) presenting for routine care, including newly diagnosed and those returning to care, and reporting no ART use within 90 days, was enrolled in a clinic‐based cross‐sectional study of TB prevalence which tested for TB using sputum and urine‐based TB tests in two clinics in KwaZulu‐Natal, South Africa. CD4 counts were determined at the time of ART initiation, per national guidelines. A novel urine‐based lateral flow assay (LFA) which detects TFV ingested within the past 4–7 days was used to assess ART use from thawed urine samples, which were collected concurrently with the self‐report assessment. Conditional logistic regression models assessed predictors of ART non‐disclosure.

**Results:**

Between 12/2021 and 5/2024, 404 PWH (40% male) reporting no recent ART use presented for ART initiation. TB testing identified 14 (3%) PWH with undiagnosed TB. Seventy‐nine (20%) had detectable TFV in urine indicating undisclosed ART use, with a median CD4 count of 466 cells/mm^3^ (IQR 277–625) compared to 322 cells/mm^3^ (IQR 175–490, *p* = 0.001) in those without undisclosed ART use. In a multivariable model, undisclosed ART use was associated with older age, rural clinic site, higher CD4 count and having active TB, but not with gender, education or employment.

**Conclusions:**

Among people presenting for HIV treatment initiation, 20% had evidence of ART use within 4–7 days by TFV urine LFA testing. Integration of point‐of‐care urine TFV assays into DSD models of HIV care may support providers to engage PWH about treatment challenges, address potential barriers to disclosure and facilitate seamless transfers between clinics. If successful, this strategy may reduce duplicative care entries and promote more efficient use of resources.

## 1 INTRODUCTION

Following its launch in 2003, South Africa's antiretroviral therapy (ART) programme has been successful, with over five million people living with HIV (PWH) initiated on treatment by 2022, resulting in a decline in HIV‐related deaths and an improved quality of life [1, 2]. Timely identification of individuals with advanced HIV disease (AHD) is crucial for optimizing care and treatment outcomes [3]. AHD, defined as HIV with a CD4 count less than 200 cells/mm^3^, has significant implications for patient prognosis, susceptibility to coinfections, and the need for additional screening and prophylaxis, in addition to ART [3].

Upon entry or re‐entry into HIV care, around 30% of individuals are found to have AHD, highlighting the importance of CD4 testing for persons not taking ART as a standard of care [4]. PWH who are not on ART are at a higher risk for tuberculosis (TB) disease due to compromised immune function [5]. TB screening is also crucial in HIV care, particularly during ART initiation/re‐initiation to mitigate the risk of immune reconstitution inflammatory syndrome, and is recommended annually for PWH who are stable on ART [6]. However, for PWH stable on ART, continued CD4 monitoring offers little benefit and is not cost‐effective [7].

In South Africa, the prevalence of undiagnosed TB among PWH not on ART is estimated to be between 5% and 10% [8, 9]. An interim analysis of our cohort study conducting intensive TB screening among PWH‐initiating ART revealed an unexpectedly low prevalence of undiagnosed TB. Only one of the first 100 enrolled PWH reporting no ART use had TB, prompting further investigation to explain this striking finding.

Undisclosed ART use, where PWH may access and ingest ART without reporting this to healthcare providers, has been identified in public health clinic settings in South Africa [10, 11] and is linked with administrative challenges faced by migrants when changing health facilities, as well as individuals who change clinics due to stigma or fear of reprimand for missed visits [12, 13]. Undisclosed ART use may lead to unnecessary HIV counselling and testing, inappropriate laboratory testing and inefficient use of healthcare resources. Detecting ART use in PWH who self‐report not taking ART is challenging, especially in settings where frequent viral load monitoring is restricted due to cost and the electronic data systems between health facilities are not linked, allowing for people to attend multiple clinics without detection.

Our group recently developed a point‐of‐care (POC) urine‐based tenofovir (TFV) immunoassay which detects TFV ingested in the last 4 days [14, 15, 16, 17]. We employed this test to estimate the prevalence of undisclosed ART use in a cohort of PWH in South Africa recruited for a TB prevalence study.

## 2 METHODS

### 2.1 Study design

We conducted a cross‐sectional study assessing undisclosed ART use nested within the DROP‐TB study, a cohort study evaluating TB prevalence and a novel urine lipoarabinomannan (LAM) TB diagnostic test [18] in South Africa. The DROP‐TB study enrolled PWH not taking ART from two public health clinics (rural and peri‐urban) in the uMkhanyakude district in KwaZulu‐Natal, South Africa. These are Department of Health primary healthcare clinics, where professional nurses provide basic primary care services, including HIV and TB testing, at no cost to the user [19]. DROP‐TB eligibility criteria included: (1) age ≥18 years; (2) a confirmed positive HIV test; (3) ART‐naïve or self‐reporting not taking ART in the prior 90 days; (4) presented to clinic for HIV testing to initiate or re‐initiate ART; and (4) did not receive TB treatment in the prior 90 days. ART use was initially assessed by the patient self‐report to the counsellor conducting the HIV test. Persons presenting to clinics for HIV testing were informed of the opportunity to participate in DROP‐TB only after initial eligibility was established, to minimize social desirability bias in self‐reporting of ART use to participate in the study.

### 2.2 Study procedures

Participants enrolling in DROP‐TB between December 2021 and May 2024 underwent a baseline clinical examination, answered standardized questions about TB and HIV history, and provided samples for routine laboratory investigations including TB diagnostic testing (performed through the National Health Laboratory Service), and research‐related testing including TB reference testing with TB culture and investigational TB assays (performed in the clinical laboratory at AHRI [20]). At the baseline visit, participants provided blood for CD4 testing, sputum for Xpert MTB/RIF Ultra (Cepheid, Sunnyvale, CA), MGIT liquid TB culture (Becton‐Dickinson, Franklin Lakes, NJ) and urine for both lateral flow LAM (LF‐LAM, Abbott) testing and the novel urine‐based LAM test. Urine remaining after DROP‐TB testing was frozen and stored at −80°C. Participants received reimbursement of ZAR 150 (USD 8.50) for participation. A *positive (prevalent) TB result* was defined as a positive sputum Xpert Ultra or TB culture, or initiation of TB treatment by a Department of Health clinician within 3 months of enrolment.

We tested thawed urine samples with the urine‐based lateral flow assay (LFA) for TFV. Urine samples were tested retrospectively as a research procedure by study laboratory personnel; results were not provided to participants or their providers. The urine TFV LFA is a low‐cost POC test that can identify the presence of TFV in urine within a timeframe of 4–7 days of ingestion. The test is 96% sensitive and 100% specific for TFV detection at concentrations >1500 ng/ml compared to the gold standard of liquid chromatography/tandem mass spectrometry (LC‐MS/MS) and can be easily implemented by healthcare personnel in real‐time [16]. The LFA has comparable accuracy in testing fresh versus frozen urine samples [16]. All urine samples were tested after being thawed to room temperature, consistent with laboratory protocol. We defined *undisclosed ART use* as a positive urine TFV LFA result in a DROP‐TB participant, since all participants reported no ART use within the past 90 days.

### 2.3 Statistical analysis

We assessed the proportion of individuals with undisclosed ART use according to the TFV LFA test. Descriptive statistics compared demographics and characteristics in those with a positive urine TFV assay to those without. We compared the proportions of categorical data with Chi‐squared tests and medians of continuous data with *t*‐tests. We employed logistic regression models to evaluate predictors of ART non‐disclosure and the association between ART non‐disclosure and TB. We fitted a multivariable logistic regression model including variables of *a priori* interest (age, sex) and included potential explanatory variables found to be significant in bivariate analyses with a *p*‐value < 0.25. Statistically significant *p*‐values in the final model were defined as less than 0.05.

### 2.4 Ethics

The study was approved by UKZN BREC/00001174/2020 and UW IRB STUDY00000124. All participants provided written informed consent to participate and for future testing of stored samples.

## 3 RESULTS

We tested stored frozen urine samples from 404 PWH enrolled in the DROP‐TB study who presented for ART initiation or re‐initiation. All participants had a positive HIV rapid test, confirmed using the standard testing algorithm, from one of two study recruitment clinics in KwaZulu‐Natal between December 2021 and May 2024. Among PWH with urine tested for TFV, 242 (60%) were women, the median age was 32 (IQR 26–39) years, and the median CD4 count was 345 cells/mm^3^ (IQR 181–510) (Table 1). Fourteen (3%) participants tested positive for TB (10 men), of whom all had at least one TB symptom. Most participants (*N* = 341, 84%) had a secondary level of education or higher, were financially dependent on social grants (*N* = 249, 61%) and were in a relationship (*N* = 288, 71%). More people attended the urban clinic (67% vs. 33%) and most (75%) did not smoke tobacco.

**Table 1:** Characteristics of PWH reporting no ART use, presenting for ART initiation, at two clinics in South Africa

	Total cohort	ART present on LFA	ART not present on LFA	
Characteristics	*N*=404	*N*=79 (20%)	*N*=325 (80%)	*p*‐value
**Gender, *N* (%)**				
Female	241/404 (60%)	45 (57%)	196 (60%)	
Male	163/404 (40%)	34 (43%)	129 (40%)	0.57
**Age, *N* (%): median (IQR)**	32 (25−38)	37 (27−39)	31 (24−36)	<0.001
18−29	148/404 (37%)	16 (20%)	132 (41%)	
30−49	235/404 (58%)	53 (67%)	182 (56%)	
>50	21/404 (5%)	10 (13%)	11 (3%)	<0.001
**Facility location, *N* (%)**				
Peri‐urban clinic	271/404 (67%)	36 (46%)	235 (72%)	
Rural clinic	133/404 (33%)	43 (54%)	90 (28%)	<0.001
**CD4 count, *N* (%): median (IQR)**	345 (181−510)	466 (277−625)	322 (175−490)	<0.001
0−200	113/398 (28%)	14/78 (18%)	99/320 (31%)	
200−350	90/398 (23%)	11/78 (14%)	79/320 (25%)	
350−500	84/398 (21%)	16/78 (21%)	68/320 (21%)	
>=500	111/398 (28%)	37/78 (47%)	74/320 (23%)	<0.001
**Highest education, *N* (%)**				
Primary	48/404 (12%)	18 (23%)	30 (9%)	
Secondary	341/404 (84%)	57 (72%)	284 (87%)	
Tertiary	3/404 (1%)	0 (0%)	3 (1%)	
Unknown	12/404 (3%)	4 (5%)	8 (3%)	0.003
**Source of income, *N* (%)**				
Employed, formal sector	86/404 (21%)	13 (16%)	73 (22%)	
Employed, informal sector	52/404 (13%)	11 (14%)	41 (13%)	
Student	16/404 (4%)	2 (3%)	14 (4%)	
Unemployed, receives social grant	90/404 (22%)	22 (28%)	68 (28%)	
Unemployed, dependent on others’ grants	158/404 (39%)	31 (39%)	127 (39%)	
Other	2/404 (1%)	0 (0%)	2 (1%)	0.61
**Smoking status, *n*/*N* (%)**				
Current	98/404 (24%)	27 (34%)	71 (22%)	
Declined to answer	1/404 (0%)	0 (0%)	1 (0%)	
Former	3/404 (1%)	0 (0%)	3 (1%)	
Never	302/404 (75%)	52 (66%)	250 (77%)	0.11
**Marital status, *N* (%)**				
In relationship	287/404 (71%)	50 (63%)	237 (73%)	
Living with a partner	33/404 (9%)	8 (10%)	25 (8%)	
Married, one spouse	22/404 (5%)	3 (4%)	19 (6%)	
Single	62/404 (15%)	18 (23%)	44 (13%)	0.15
**TB positive, *N* (%)**	14/404 (3%)	6 (8%)	8 (2%)	0.02
**Ever taken ART, *N* (%)**				
No	353/404 (87%)	57 (72%)	296 (91%)	
Yes, but not in 90 days	51/404 (13%)	22 (23%)	29 (9%)	<0.001

Abbreviations: ART, antiretroviral therapy; IQR, inter‐quartile range; LFA, lateral flow assay.

Undisclosed ART use was identified through urine TFV in 79 (20%) of participants, with 57/79 (72%) reporting to be ART‐naïve. PWH with undisclosed ART use were older (median age: 37 vs. 31 years) and had higher CD4 counts (median: 466 vs. 322 cells/µl). Among people with TB, 40% had recent TFV use (Table 1). In multivariable models, odds of undisclosed ART use increased with increasing age (aOR 2.4 [95% CI 1.31−4.38, *p* = 0.004] for ages 30–49; aOR 7.2 [95% CI 2.75−20.4, *p* = 0.001] for ages >50 vs. age <30), increasing CD4 count (aOR 3.61 [95% CI 1.78−7.01, *p* = 0.001] for CD4 ≥500 vs. CD4 <200), attendance at the rural clinic versus urban (aOR 3.13 [95% CI 1.89−5.18, *p* = 0.001]) and having TB (aOR 3.26 [95% CI 1.09–9.70, *p* = 0.03]) (Table 2).

**Table 2:** Predictors of undisclosed ART use in PWH

	*n*/*N* (%)	Adjusted odds ratio (95% CI)	*p*‐value
**Gender**			
Female	242/404 (60)	ref	0.57
Male	163/404 (40)	1.15 (0.70−1.89)	
**Age**			
18−29	148/404 (37)	ref	
30−49	235/404 (58)	2.40 (1.31−4.38)	0.004
>50	21/404 (5)	7.5 (2.75−20.41)	<0.001
**CD4 count (cells/mm^3^)**			
0−200	113/398 (28)	ref	
200−350	91/398 (23)	0.97 (0.41−2.25)	0.94
350−500 > = 500	84/398 (21) 111/398 (28)	1.66 (0.76−3.63) 3.61 (1.78−7.01)	0.20 <0.001
**Clinic location**:			
Peri‐urban	272/404 (67)	ref	
Rural	133/404 (33)	3.13 (1.89−5.18)	<0.001
**TB status**			
Negative	391/404 (97)	ref	
Positive	14/404 (3)	3.26 (1.09–9.70)	0.03

## 4 DISCUSSION

Using a novel urine TFV LFA, we found that 20% of PWH presenting to two clinics for ART initiation and re‐initiation had undisclosed ART use. This is consistent with previous studies which suggest that a substantial number of PWH may not report ART use at public health clinics in South Africa [10, 11]. Interestingly, 72% (57/79) of those with undisclosed ART use reported to be ART‐naïve, highlighting the importance of targeting this population for TFV urine testing during HIV screening. However, the urine test should not be limited to this group, as it may miss a substantial proportion of individuals who are using ART but not disclosing.

Undisclosed ART use was associated with older age, higher CD4 counts, attending the rural clinic and having active TB. One possible explanation for these associations is that individuals who do not disclose their ART use may be presenting to clinics while virally suppressed and stable on treatment. Older age and higher CD4 counts may reflect a longer duration of ART use [21]. The association with rural sites may be driven by limited job opportunities in rural areas: In public health clinics, where clients do not have portable electronic medical records, health workers may request a physical transfer letter from the previous clinic. PWH presenting to a clinic to continue stable ART previously received elsewhere may be declined enrolment without a transfer letter from the previous clinic [22]. In contrast, no documentation is required to initiate ART. Individuals from rural areas may migrate for employment and establish ART care locally, but upon returning home, may not disclose their ART use to avoid the cost and inconvenience of obtaining transfer letters from their ART site, preferring to present as ART‐naïve [11, 12, 13, 22]. Contrary to our initial hypothesis, we found a significant positive association between active TB and undisclosed ART use. Since most of those with TB were men (70%), the association may reflect employment‐related migration of men. This unexpected finding underscores the importance of routine TB screening at every clinic visit, regardless of ART status.

South Africa has the world's largest ART programme and non‐disclosure of ART to clinics will result in higher costs from unnecessary testing, treatment and personnel time spent on counselling. Reducing non‐disclosures could optimize the use of resources for testing and clinical care. The high frequency of non‐disclosure highlights the need for person‐friendly differentiated service delivery (DSD) approaches that cater to the unique needs of individuals who may be accessing ART without disclosing to healthcare providers, particularly those who are highly mobile and need to change clinics frequently [12, 13]. By addressing the root causes of non‐disclosure, DSD models can provide tailored support and monitoring strategies to improve patient outcomes and optimize healthcare resource usage [23], as the current clinic transfer practice is not person‐centred. People who conceal ART are mobile populations who relocate for work, family obligations and stigma linked with experiences at prior clinics [12]. The need for flexible and compassionate approaches that consider unique circumstances should be encouraged in healthcare.

Resources utilized at initiation visits are more intensive compared to those at continuation visits. During initiation, comprehensive diagnostic tests, extensive counselling, and education on HIV and ART are provided, along with initial treatment planning and close monitoring for potential drug interactions and side effects. In contrast, continuation visits focus on viral load monitoring, adherence support and less frequent medication refills (Table 3). Therefore, accurately identifying true ART initiators versus those re‐entering care after undocumented transfers is crucial [24]. At present, there are greater administrative challenges in confirming the continuation of care than in initiating or re‐initiating it [12]. Our proposal is to use the test as a tool to identify individuals who need continuation care, while an integrated system with unique identifiers is implemented. The introduction of this test must be accompanied by extensive training, and removal of burdensome administrative requirements of transfer letters, to ensure it does not become another tool for stigmatization. The goal is not to make this test a permanent fixture in care, but to use it as an interim solution to address non‐disclosure until an integrated system is in place.

**Table 3:** Difference between resources used and client follow‐up burden/frequency during ART initiation versus ART continuation visit at clinics in South Africa

Resources	ART initiation	ART continuation
**HIV diagnostics**	HIV rapid testing	No testing
**Laboratory tests**	CD4, CrCl, TB lcNAAT, Hb, HBsAg and CrAg if CD4 < 100 cells/µl No VL testing	VL at M3, M10, M22 and yearly TB lcNAAT yearly
**Counselling**	HIV, ART and adherence	Support
**Medication**	Initial treatment education and planning, 1M supply dispensed	Medication refills 3−6M supply dispensed
**Screening**	TB, STIs, pregnancy, NCDs, cervical cancer, mental health issues and meningitis	TB symptoms
**Additional support**	Social work, mental health and so on	Less intensive support services
**Monitoring**	Close monitoring for potential drug interactions and toxicity	Ongoing care and maintenance
**Examination**	Full physical examination to determine clinical stage	No physical examination
**Return frequency**	1 week follow‐up, 1M follow‐up, 3M follow‐up	6M follow‐up

*Note*: Summarized from the 2023 South African ART clinical guidelines [22].

Abbreviations: CrAg, cryptococcal antigen; CrCl, creatinine clearance; Hb, haemoglobin; HBsAg, hepatitis B surface antigen; M, month; TB lcNAAT, low‐complexity nucleic acid amplification test, for example Xpert MTB/RIF Ultra; VL, viral load.

There were several limitations to this study. Only two health facilities were included in the analysis, which limits the generalizability of the results. The urine LFA detects TFV only and not other antiretrovirals. However, TFV is part of the first‐line fixed‐dose ART regimen used in South Africa and much of the world—tenofovir‐lamivudine‐dolutegravir—so it is a robust indicator of ART use. Although TFV is also a component of oral Pre‐Exposure Prophylaxis (PrEP) for HIV prevention, PrEP use is uncommon in the study area [25, 26] and this study limited eligibility to those with a positive HIV test. This indicates that a positive urine TFV represented ART use, not PrEP.

Our study is unique in reporting on undisclosed ART use at public health clinics in South Africa in using an objective metric of ART exposure, verifying use with an LFA designed to be used in clinic settings. Other studies investigating ART non‐disclosure used laboratory‐based testing to verify ART exposure, which is expensive and requires laboratory‐based personnel [10, 11, 16]. A POC urine LFA costs less than USD 2 per test and can be performed by healthcare personnel in the clinic. Urine TFV LFAs are highly sensitive, easy to use and can be used as an immediate solution to identify undisclosed ART use at clinics in South Africa, since PWH may be disincentivized to disclose ART use with current clinic practices. Given the high proportion of undisclosed ART use detected in our study, we recommend using urine TFV LFAs during clinic‐based HIV testing in South Africa. PWH found to have TFV in urine can be asked, in a non‐judgemental manner, about ART use and reasons for non‐disclosure. Clinicians can then determine whether ART continuation procedures may be appropriate (instead of initiation) and facilitate a formal transfer from the previous clinic, streamlining care and avoiding redundant testing. To effectively integrate POC urine LFA into routine HIV testing, healthcare workers would require training, not only about the challenges faced by individuals with undisclosed ART, but also how to respond in a welcoming way when identifying ART use through a test result, ultimately enabling patient‐centred care and support.

## 5 CONCLUSIONS

By utilizing POC urine LFA testing for TFV, individuals with HIV on ART can be identified, allowing for appropriate DSD referrals as or until clinic practices are transformed to incentivize disclosure. This approach may improve the accuracy of national HIV treatment retention metrics and optimize resource allocation, leading to a more robust South African ART programme.

## COMPETING INTERESTS

1

AES receives grant funding from Merck as a clinical trial investigator.

### AUTHORS’ CONTRIBUTIONS

NS, MG and AES designed the study. NS and NP collected the data. TS, SC and MZ tested the samples. NS and AES analysed the data. NS and AES drafted the manuscript. All authors contributed to the revisions and content of the final manuscript.

## FUNDING

The study was funded by the National Institutes of Health (NIH)/National Institute of Allergy and Infectious Diseases (NIAID) (K23 AI140918), with additional support from K23 MH122286 (MAS) and 2R01AI143340 (MAS, MG). MJS acknowledges additional support from K24 HL166024.

## Data Availability

The data that support the findings of this study are available on request from the corresponding author. The data are not publicly available due to privacy or ethical restrictions.
